# Uncommon Metastasis of a Large-Cell Neuroendocrine Carcinoma From the Lungs to the Buccal Palatal Region

**DOI:** 10.7759/cureus.67469

**Published:** 2024-08-22

**Authors:** Jihane Derfoufi, Fatima Rezzoug, Meryem El Jarroudi, Karich Nassira, Ouissam Al Jarroudi, Sami Aziz Brahmi, Said Afqir

**Affiliations:** 1 Medical Oncology, Mohammed VI University Hospital, Faculty of Medicine and Pharmacy, Mohamed I University, Oujda, MAR; 2 Pathology, Mohammed VI University Hospital, Oujda, MAR; 3 Medical Oncology, Mohammed VI University Hospital, Oujda, MAR

**Keywords:** systemic chemotherapy, case report, metastatic non-small cell lung cancer, palatal metastasis, lung neuroendocrine tumor

## Abstract

The metastasis of a primary lung tumor to the mouth cavity is a rare occurrence. In addition, the occurrence of neuroendocrine bronchial carcinoma with large cells is uncommon. When metastases are not possible to surgically remove, the conventional treatment for large-cell neuroendocrine tumors (LCNET) is still used. The etiology of these metastases remains inadequately comprehended, rendering their administration very intricate. The oncologist at this institution must possess a comprehensive comprehension of how to effectively oversee the patient's quality of life to guarantee the uninterrupted progression of therapy. This paper is a case study of a 51-year-old male patient who was hospitalized due to a severe dry cough and dysphonia that began two months prior to seeking medical consultation. Gingival hyperplasia was diagnosed during a clinical examination. The diagnosis of LCNET (carcinoma of the lung) was determined after a thorough etiological investigation utilizing gingival samples and pulmonary tissue. The objective of this study was to provide a description of our case, conduct an analysis of the response to therapy, and make a contribution to the current body of research. The purpose was to encourage more investigation into this type of metastasis, aiming to get a deeper comprehension of the mechanisms behind the metastatic spread and assess its predictive significance in future instances.

## Introduction

Lung cancer is widely acknowledged as one of the most often diagnosed forms of cancer globally. It is the primary cause of cancer-related fatalities for both males and females, with around 2.20 million cases and 1.79 million deaths recorded each year [1]. Neuroendocrine tumors (NECs) are a heterogeneous collection of growths that arise from neuroendocrine cells [2]. Pulmonary large-cell neuroendocrine carcinoma (LCNEC) is a rare kind of lung cancer. The tumor originates from argyrophilic cells in the lung. The tumor has characteristic shape and differentiation characteristics of a neuroendocrine tumor [3,4]. LCNEC is a rare and very aggressive form of lung cancer. The first study was authored by Travis et al. [5]. Nevertheless, our categorization methods have since seen modifications and progress. The World Health Organization (WHO) acknowledged large-cell neuroendocrine carcinoma (LCNEC) as a subtype of non-small cell lung cancer (NSCLC) called large-cell carcinoma (LCC) in 1999 and 2004 [6]. However, in 2015, the World Health Organization (WHO) changed the classification of LCNEC from large-cell carcinoma to neuroendocrine carcinoma [7]. In the latest update of the WHO classification in 2021, LCNEC remains classified as a neuroendocrine carcinoma. The focus is on using molecular type to assist in diagnosing the condition and making treatment decisions [8]. In addition, it is worth noting that patients with LCNEC have a demographic profile that is characterized by a higher prevalence among males, older adults, and heavy smokers [9]. Non-smoking females are seldom affected by this form of cancer. The age at which a diagnosis is often made is around 60 years, as reported in the study by Ichiki et al. [9]. The spread of cancer in LCNEC often affects the liver, bone, brain, adrenal gland, lung, pleura, and lymph nodes outside the chest [10]. Nevertheless, there have been infrequent reports of metastatic large-cell neuroendocrine carcinoma (LCNEC) developing in the oral and maxillofacial areas [11]. In this case, we presented a unique occurrence of a metastasis from a large-cell neuroendocrine carcinoma of the lungs to the buccal palatal region in a 51-year-old male patient. A confrontation between the two specimens was conducted to illustrate their main lung origin and prominent big-cell neuroendocrine features.

## Case presentation

We present a male patient who is 51 years old, has a history of heavy smoking equivalent to 40 pack-years, and has a family history of his father's death from lung cancer. The patient was admitted due to a severe dry cough and dysphonia that began two months prior to seeking medical attention. The clinical assessment reveals a patient who is in a satisfactory overall state of health, with stage two dyspnea, dysphonia, and dysphagia for solid food. No clinical evidence of superior vena cava syndrome or symptoms of cardiovascular overload, nor clinical evidence of carcinoid disease or ectopic Cushing's syndrome were seen. Upon oral cavity inspection, a palatal growth was seen. No abnormalities were found throughout the remaining systemic testing.

**Figure 1:**
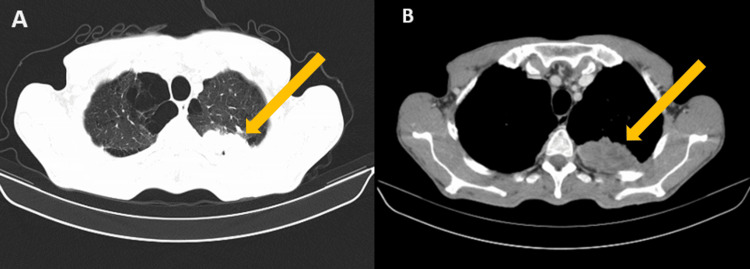
Transverse radiological section showing a large, tumor-like heterogeneous mass in the lung.

At first, a full-body CT-CSAN was performed and revealed bilateral pulmonary emphysema, a large tumor-like heterogeneous mass measuring 125x96x108 mm with close vascular contact, on the upper mediastinum. A second mass was found in the left upper lip, measuring 36x70 mm, in addition to multiple bilateral metastatic-looking lymph nodes. No other distant metastasis was found other than bilateral adrenal lesions (Figures 1A, 1B).

**Figure 2:**
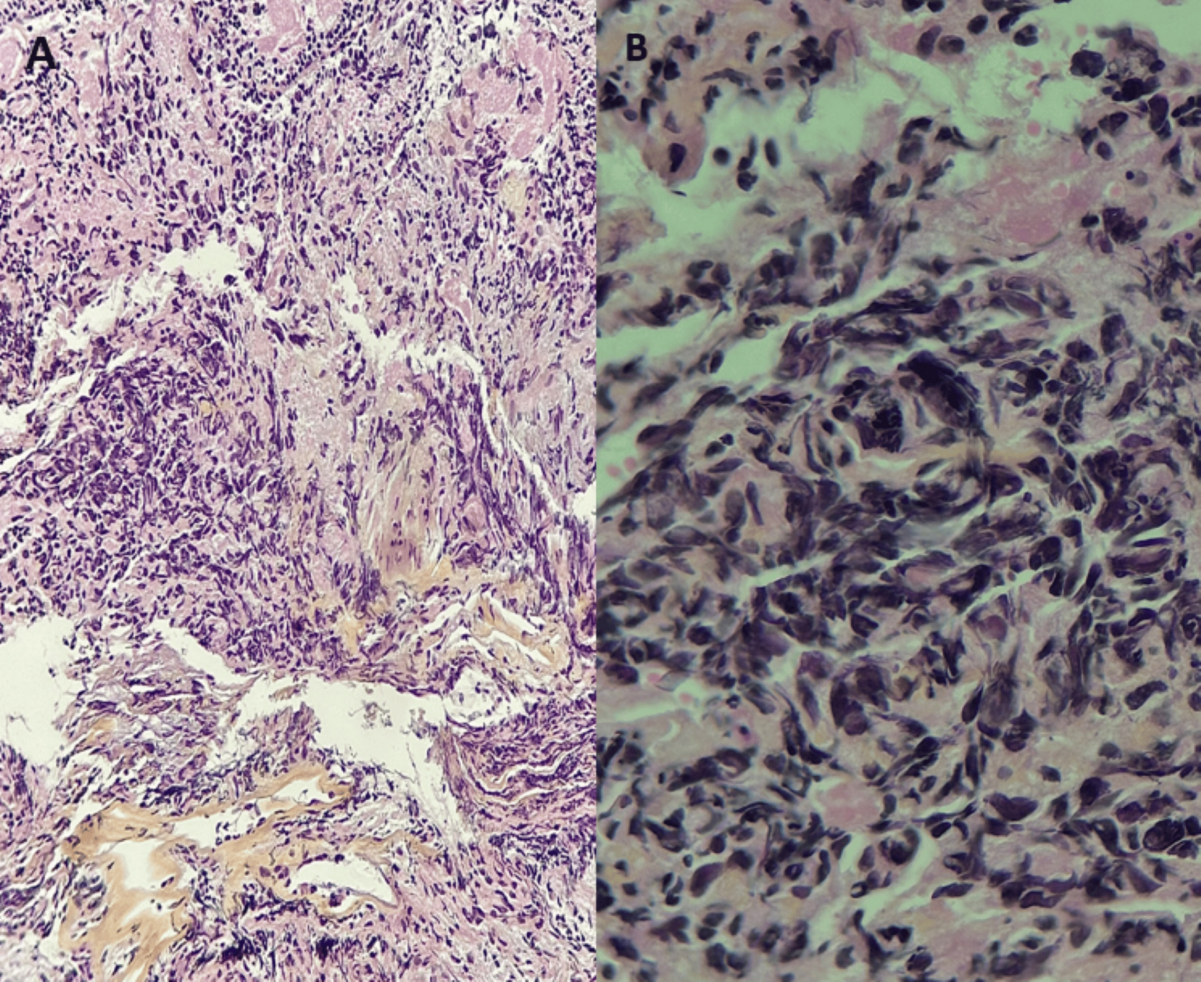
Histology shows sheets of gigantic, atypical tumor cells with nucleolated nuclei and vesicular chromatin compressing into regions. Several mitotic figures appear. (A) Staining with hematoxylin-eosin saffron at 20x magnification (HES x20). (B) Staining with hematoxylin-eosin saffron at 40x magnification (HES x40).

Anatomopathological and immunohistochemical study was performed at first on a pulmonary specimen and revealed a large-cell neuroendocrine pulmonary carcinoma expressing chromogranin A and synaptophysin with a very high proliferation index (Ki67) of 90% (Figures 2A, 2B).

**Figure 3:**
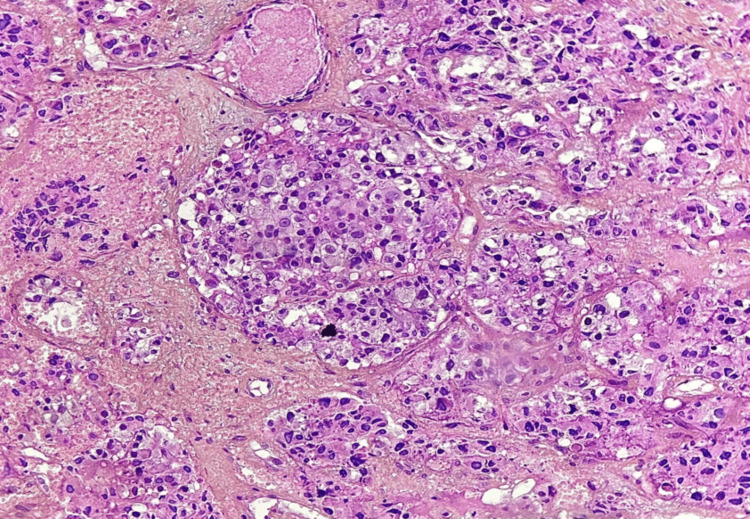
Histological image showing tumor growth in masses and nucleus. Large tumor cells have finely nucleated irregular nucleus and eosinophilic cytoplasm. Staining with hematoxylin-eosin saffron at 50x magnification (HES x50).

A second specimen was taken from the palatal lesion, leading to the same results (Figure 3). The pathological and radiological findings point out a primary pulmonary origin. A confrontation between the two specimens was performed, confirming their primary lung origin and large-cell neuroendocrine features.

After a multidisciplinary meeting and in accordance with the latest recommendations and oncological guidelines, as well as considering the available resources, the chosen cytotoxic treatment was first-line chemotherapy consisting of cisplatin 100 mg/m² on day one and etoposide 100 mg/m² for three days, administered every three weeks. Our patient received three cycles of chemotherapy. The evolution of the patient's illness was characterized by a clear clinical progression and a change in his overall state, as shown by an Eastern Cooperative Oncology Group score of two (ECOG=2). There was a deterioration of dysphagia and dysphonia, accompanied by a dry cough and paraneoplastic syndrome as revealed by blood tests. A radiological evaluation was performed, favoring a progression of the primary tumor and the metastasis. The patient was programmed for second-line chemotherapy using weekly paclitaxel, but we lost contact with the patient, the patient was reported dead a few days later.

## Discussion

Lung cancer is quite frequent and is often discovered at the advanced stage, yet palatal localization of primary lung cancer is rare. It is the second most frequent tumor and the most common cause of mortality due to cancer in the world. Mainly due to tobacco use in around 80% [12]. Lung cancer can often be lethal due to a late diagnosis. The prognosis of these patients depends on both initial TNM staging and tumor histopathology [13]. Lung cancer is classically divided into two following types: non-small cell lung cancer (81% of cases) and small cell lung cancer (14% of cases) [14]. Five-year survival is less than 1% for extensive small cell lung cancer (SCLC) and varies from 0% to 10% for non-small cell lung cancer (NSCLC) [15]. In this study, we will focus only on large-cell neuroendocrine lung carcinoma, which describes the case of our patient.

Large-cell neuroendocrine carcinoma (LCNEC) of the lung is a rare but aggressive tumor and has a bad prognosis. It is generally diagnosed late as an advanced illness. It accounts for 40% of resected lung neuroendocrine tumors and 1-3% of all lung malignancies [3]. On the basis of their neuroendocrine morphology and immunohistochemical features, large-cell neuroendocrine carcinomas can be identified as belonging to one of four different phenotypes. It is more frequent in males, occurring at a ratio of four to one compared to females, and the average age at which symptoms first manifest is 66 years compared to the average age of females. The majority of those who were diagnosed with LCNEC had a smoking history, which ranged from 85% to 98% [16,17]. The clinical presentation and medical history of LCNEC closely mimic those of small cell lung cancer (SCLC) [16]. Lung large-cell neuroendocrine carcinomas (LCNECs) mainly develop in the peripheral area of the lungs, specifically in the upper lobes. Patients can show up with no or non-specific symptoms. Paraneoplastic syndromes associated with lung malignancies are rare [18]. This is a group of lung tumors that exhibit a neuroendocrine phenotype under light microscopy but do not fit into any of the three recognized neuroendocrine tumor categories. Radiographic findings, clinical examination, and microscopic evaluation are used to make a diagnosis in these cases. Imaging studies like computed tomography (CT), positron emission tomography (PET), and routine chest radiography are recommended, and these tests usually show a mass that is 3-4 cm in size. Microscopic examination can help establish morphological features and aid in diagnosis, while immunohistochemistry enhances the diagnostic process. Immunoperoxidase staining using neuroendocrine markers (CD56, chromogranin, and synaptophysin) and TTF-1 are useful immunohistochemical techniques [19]. SCLC and several NSCLC subtypes are the primary differential diagnoses for large-cell neuroendocrine carcinoma (LCNEC). High-grade neuroendocrine carcinomas, such as small cell lung carcinoma (SCLC) and large-cell neuroendocrine carcinoma (LCNEC), have similar immunohistochemical characteristics. The primary differences are cytological, such as cell size, cytoplasm, and nucleoli. The retinoblastoma protein 1 (RB1) expression is absent in over 95% of small cell lung cancers (SCLCs) but only 50% of big cell neuroendocrine tumors. Thus, LCNEC is more likely to be detected in those with normal RB1 expression when differential diagnosis is difficult [20].

Understanding the metastatic process requires a deep understanding of the intricate biological mechanisms involved. These mechanisms include the detachment of cells from their surrounding environment, the regulation of cell motility and invasion, the ability to survive and proliferate, and the clever evasion of the immune system [21]. The spread of cancer from one organ to another is not an uncontrolled occurrence but rather a controlled, site-specific process, according to extensive research [21]. Metastatic tumor cells rarely spread in the mouth, and when they do, it's usually a sign of an illness that has propagated widely [21]. During the literature search from 1992 to 2006, a total of 93 articles were found, which reported 126 cases of metastatic lesions to the oral cavity. Out of these cases, 61 involved the mucosa, and 65 involved the jawbones. Most cases were reported as individual case reports or small series of cases [22]. According to Batson, the valveless vertebral venous plexus serves as a way to bypass filtration through the lungs. When there is an increase in intrathoracic pressure, blood flow is directed into this system from the caval and azygous venous systems. This mechanism explains the increased distribution of the axial skeleton and head and neck metastasis [22]. In the oral soft tissues, the attached gingiva is frequently targeted for metastatic colonization. There is a potential connection between inflammation and the migration of metastatic cells to the attached gingiva [21]. Diagnosing a metastatic lesion in the oral region can be quite challenging for both clinicians and pathologists. It requires careful recognition of the lesion as metastatic and determining its site of origin. When a metastatic lesion appears in the oral cavity, it can be misleading and may result in a misdiagnosis of a benign condition. Therefore, it is crucial to perform a biopsy in cases where the clinical presentation is atypical, particularly in patients who already have a confirmed malignant disease [22].

LCNEC is a type of non-small cell lung cancer because its tumors behave biologically similarly to SCLC tumors, so the choice of treatment for LCNEC patients is still up for debate [20]. To date, there is no consensus on treatment for large-cell neuroendocrine carcinoma (LCNEC). When it comes to the treatment of advanced pulmonary large-cell neuroendocrine carcinoma, platinum-based chemotherapy has shown some promising benefits in the short term. The full approach to treating LCNEC includes a number of different treatment modalities, including surgery, radiation therapy, chemotherapy, immunotherapy, and targeted therapy [23]. When treating advanced pulmonary large-cell neuroendocrine carcinoma, platinum-based chemotherapy does show some short-term benefits. Continuous intravenous infusion of cisplatin and etoposide chemotherapy is the gold standard for treating advanced SCLC [20]. Treatment with platinum and etoposide resulted in a significant response rate for LCNEC patients. Also, in their phase II trial, Niho et al. reviewed 30 patients with LCNEC who were treated with irinotecan and cisplatin. They reported a response rate of 46.7% [24]. Peri and Fazio administered everolimus as the initial treatment for advanced and non-functional lung neuroendocrine tumors (LNETs) [25]. In a phase II study, chemotherapy-naive stage IV LCNEC patients received 5 mg of everolimus daily, paclitaxel (175 mg/m^2^), and carboplatin AUC 5 every three weeks for up to four cycles. Maintenance everolimus at 5 mg/day until progression. ORR was 45%, disease control was 74%, median PFS was 4.4 months, and OS was 9.9 months. Kaplan-Meier analysis indicated a 76% three-month progression-free survival (PFS) rate, the key endpoint [25]. Christopoulos et al. separated patients into three groups as follows: group I received gemcitabine, docetaxel, paclitaxel, or vinorelbine; group II received pemetrexed alone; and group III received etoposide treatment. LCNEC patients with group I treatment had a longer overall survival (OS) than groups II and III [25]. Furthermore, immune checkpoint inhibitors (ICIs) targeting programmed cell death protein 1 (PD-1) and its ligand (PD-L1) are commonly used to treat advanced non-small cell lung cancer (NSCLC) and small cell lung cancer (SCLC) [20]. Researchers are investigating novel chemotherapeutic options for the treatment of large-cell neuroendocrine carcinoma (LCNEC).

However, in our case, surgical intervention was not indicated. The patient had received three cycles of cisplatin and etoposide treatment, spanning a duration of three months. Unfortunately, the intraoral lesion rapidly progressed clinically. The patient's visceral lesions remained stable, according to the CT scan. However, the patient passed away a few weeks after completing the third cycle. Several studies have indicated that the prognostic characteristics of large-cell neuroendocrine carcinoma (LCNEC) are associated with age, gender, therapy, tumor size, metastasis, and the number of positive lymph nodes.

## Conclusions

We highlight a very aggressive illness that has a bad prognosis by presenting a report in which we demonstrate the uncommon occurrence of palatine metastasis that originated from neuroendocrine lung cancer with big cells. In order to properly diagnose high-grade LNETs, a comprehensive histopathological analysis and a knowledge of the molecular landscape are required. This occurrence has only been observed in a small number of case reports, which highlights the necessity of doing more research in order to develop a management approach that is both more precise and more successful for this form of metastasis.
